# MiR-31 regulates the cisplatin resistance by targeting Src in gallbladder cancer

**DOI:** 10.18632/oncotarget.13067

**Published:** 2016-11-04

**Authors:** Maolan Li, Wei Chen, Hongchen Zhang, Yong Zhang, Fayong Ke, Xiangsong Wu, Yijian Zhang, Mingzhe Weng, Yingbin Liu, Wei Gong

**Affiliations:** ^1^ Department of General Surgery, Xinhua Hospital, School of Medicine, Shanghai Jiao Tong University, Shanghai 200092, China; ^2^ The Institute of Biliary Disease Research, School of Medicine, Shanghai Jiao Tong University, Shanghai 200092, China

**Keywords:** gallbladder cancer, miR-31, DDP, src, drug resistance

## Abstract

**Background:**

Gallbladder cancer (GBC) is a malignant tumor highly resistant to chemotherapy. MicroRNAs (miRNAs) are found extensively involved in modulation of carcinogenesis and chemoresistance. This study aimed to investigate cisplatin (DDP)-susceptibility regulated by expression of the miRNAs and underlying pathways in GBC.

**Results:**

The microRNA-31 (miR-31) was selected by microarray due to the biggest fold change between DDP-resistant and parental cells. Ectopic overexpression of miR-31 decreased cell proliferation, viability and invasion capacity, but promoted apoptosis in DDP-resistant cells and in xenograft tumor models. Cell apoptosis and DDP-chemosensitivity was remarkably increased by knockdown of Src proto-oncogene (Src) expression, which was subsequently reversed by rescue of Src expression in miR-31-expressing cells.

**Methods:**

The microarray was used to select the candidate miRNA in two DDP-resistant GBC cell lines. The effect of regulated expression of the miRNA on cell migration, invasion, proliferation and apoptosis was examined by wound healing, transwell assays, CCK-8 assays, colony formation and flow cytometry assays, respectively. Xenograft tumor models were used to validate the function of the downstream target.

**Conclusion:**

Our results demonstrated that miR-31reduced significantly in GBC cells rendering resistance to cisplatin, and upregulated expression of miR-31 augmented chemosensitivity, presenting a therapeutic potential to overcome drug resistance in GBC.

## INTRODUCTION

Gallbladder cancer (GBC), a highly aggressive and the most widespread neoplasm of the biliary tract, is clinically characterized by late diagnosis and high recurrence after surgery [1, 2]. Chemotherapy is administered to extend patients survival by effectively reducing tumor size and suppressing distant metastasis [3, 4]. One of the first-line chemotherapeutic agents, cisplatin (also known as DDP), has been universally found to benefit individuals with advanced, unresectable or metastatic GBC [5, 6]. However, a high resistance to DDP rapidly evolved in a substantial number of patients with GBC, becoming a crucial bottleneck of chemotherapy.

MicroRNAs (miRNAs), single-stranded RNA molecules with 19~25 nucleotides in length, are recognized as key modulators governing multiple gene expression via binding to the 3′ untranslated region (3′-UTR) of target mRNA at the posttranscriptional level [7]. A range of miRNAs with aberrant expression may function differently in tumor suppression, oncogenesis or even the evolvement of chemoresistance in various cancer types [8]. MicroRNA-31 (miR-31) has been detected in diverse tumor types, such as bladder cancer, esophageal cancer, ovarian cancer, prostate cancer and breast cancer [9–13]. But the regulative roles of miR-31in the chemoresistant GBC remains elusive.

In this study, we uncovered that miR-31was downregulated in DDP- resistant GBC cells through microarray-based screens, and demonstrated for the first time that ectopic overexpression of miR-31 conferred enhanced chemosensitivity to DDP both *in vitro* and *in vivo*. We further explored the potential involvement of miR-31/Src/Akt/Bax/Bcl-2 pathway, by which miR-31 significantly regulated the sensitivity of GBC cells to DDP. Our work may present a promising therapeutic target in the management of chemoresistance in GBC.

## RESULTS

### MiR-31 is down-regulated in DDP-resistant GBC cells

**Figure 1:**
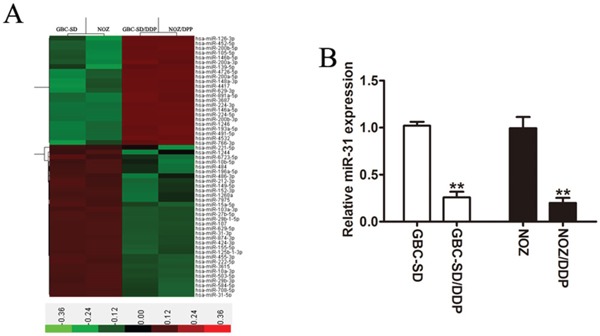
Down-regulation of miR-31 in DDP-resistant GBC cells **A.** Heatmap representation of the expression difference of miRNAs in the GBC-SD, NOZ and the DDP-resistant GBC cells. The horizontal axis signifies the expression of miRNAs, and columns represent the biological replicates. Red, high expression; green, low expression. **B.** The expression level of miR-31 detected in DDP-resistant cells (GBC-SD/DDP and NOZ/DDP cells) and in its parental cells by qRT-PCR. Data were presented as mean ± SD. **P < 0.01.

Through microarrays, we identified the miRNA most likely contributed to the acquisition of DDP-resistance in GBC cells. In a set of 47 miRNAs with expression change more than 2-fold in DDP-resistant cells compared to their parental cells, miR-31was the one with the lowest expression in DDP-resistant cells (Figure 1A). The qRT-PCR quantification analysis confirmed that the expression level of miR-31 was remarkably down-regulated in GBC-SD/DDP and NOZ/DDP cells in comparison to their parental cells (Figure 1B).

### Effect of upregulated miR-31 on DDP-sensitivity and invasion capacity of GBC-SD/DDP and NOZ/DDP cells

**Figure 2:**
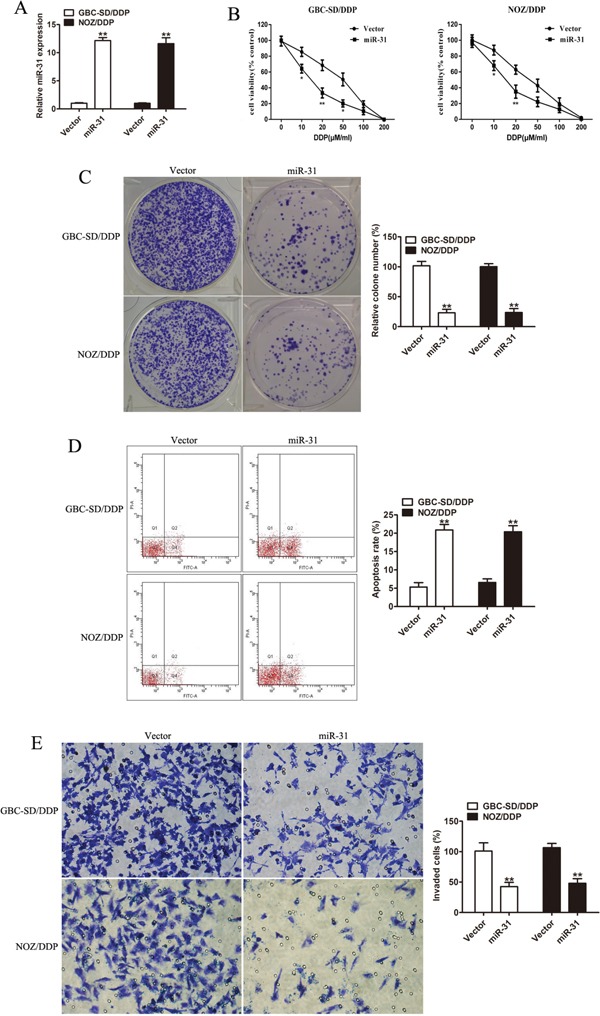
Effect of miR-31 on cisplatin sensitivity and invasion capacity of DDP-resistant cells **A.** The affirmation of the level of miR-31 mRNA expressed in GBC-SD/DDP and NOZ/DDP cells transfected with miR-31 mimic by qRT-PCR. **B.** The cell viability plotted against the concentration of DDP in GBC-SD/DPP and NOZ/DPP cells transfected with miR-31 or vector. **C.** Colony formation assay of DDP-resistant cells transfected with miR-31 or vector after exposure to DDP. **D.** The DDP-induced apoptosis rate of GBC-SD/DPP and NOZ/DPP cells transfected with miR-31 or vector analyzed by flow cytometry. **E.** The invasive ability of DDP-resistant cells transfected with miR-31 or vector after treatment with DDP in the transwell invasion assay. Data were presented as mean ± SD. *P < 0.05; **P < 0.01.

To validate the regulative role of miR-31 in modulating the sensitivity of GBC cells to DDP, the DDP-resistant cells (GBC-SD/DDP and NOZ/DDP cells) were stably transfected with miR-31 mimic or empty vector, and the transfection efficiency was affirmed by qRT-PCR (Figure 2A). Compared to the control group, the DDP-resistant cells with over-expressed miR-31 produced lower cell viability/higher DDP-sensitivity (Figure 2B), lower numbers of colonies (Figure 2C), and a higher rate of DDP-induced apoptosis (Figure 2D). Of note, the transwell invasion assay indicated that the invasive ability was crucially hindered in DDP-resistant GBC cells with overexpressed miR-31 (Figure 2E).

### Src is a direct target gene of miR-31 and inversely correlated with miR-31 in GBC patients

**Figure 3:**
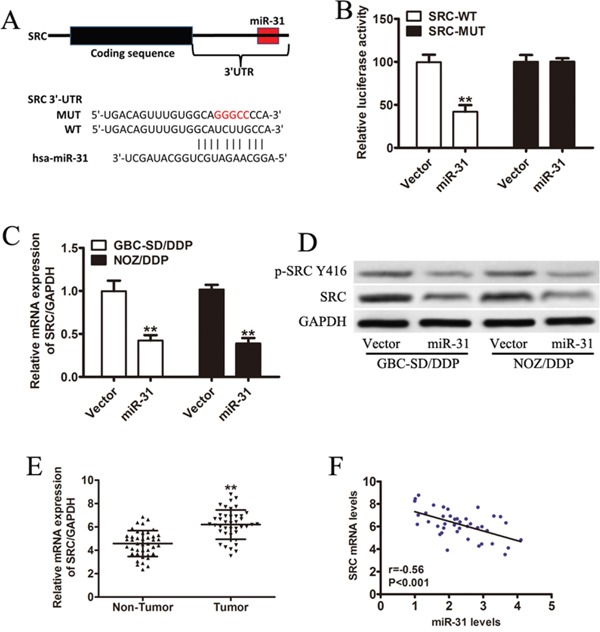
Src is a direct target gene of miR-31 and inversely correlated with miR-31 in GBC patients **A.** Sequence of the miR-31-binding site within the human Src 3′-UTR and a schematic diagram of the reporter construct showing the entire Src 3′-UTR sequence and the mutated Src 3′-UTR sequence. Src-WT: wild-type; Src-MUT: mutant type **B.** Luciferase assay of NOZ/DDP cells co-transfected with vector or miR-31 and a luciferase reporter containing the full length of Src 3′-UTR (WT) or a mutant (MUT). Luciferase activities were measured 24 hours post-transfection. Src-WT: wild-type; Src-MUT: mutant type **C.** The expression level of Src measured by qRT-PCR and normalized to GAPDH. **D.** The immunoblotting data of Src and p-Src(Y416) expression in DDP-resistant GBC cells transfected with miR-31 or vector. **E.** The mRNA expression of Src in tumor tissues and the adjacent non-tumor tissues from 41 GBC patients by qRT-PCR. **F.** An inverse correlation between miR-31 and Src mRNA expression by Pearson correlation analysis (Pearson's correlation: r= −0.56, *P*<0.001). Data were presented as mean ± SD. **P < 0.01.

To investigate the signaling network which might involve in miR-31-mediated DDP-resistance in GBC cells, we looked into the potential binding sites of miR-31 by the online miRNA target gene prediction tool (Target Scan and miRBase databases, Supplementary Table S1), and identified Src, a non-receptor tyrosine kinase known to regulate the drug-susceptibility of cancer cells, as our candidate (Figure 3A). To examine whether miR-31 directly bounds to Src, we performed luciferase reporter assay. The miR-31-transfected GBC cells co-transfected with the wild-type Src 3′UTR showed the dramatically repressed activity of the luciferase, whereas those co-transfected with mutant Src 3′UTR did not show clear changes in luciferase activity (Figure 3B). Furthermore, DDP-resistant GBC cells with ectopic overexpression of miR-31 yielded robust decreases in Src expression at both mRNA and protein levels (Figure 3C, 3D). In addition, the mRNA expression level of Src was significantly higher in tumor tissues than that in the adjacent non-tumor tissues from 41 GBC patients (Figure 3E, *P* < 0.01). Finally, as shown in Figure 3F, an inverse correlation between miR-31 and Src mRNA expression was observed in 41 GBC tissue samples (Pearson's correlation r= −0.56, *p*<0.001).

### Loss of Src restores sensitivity to DDP and reduces the invasion capacity of GBC-SD/DDP and NOZ/DDP cells

**Figure 4:**
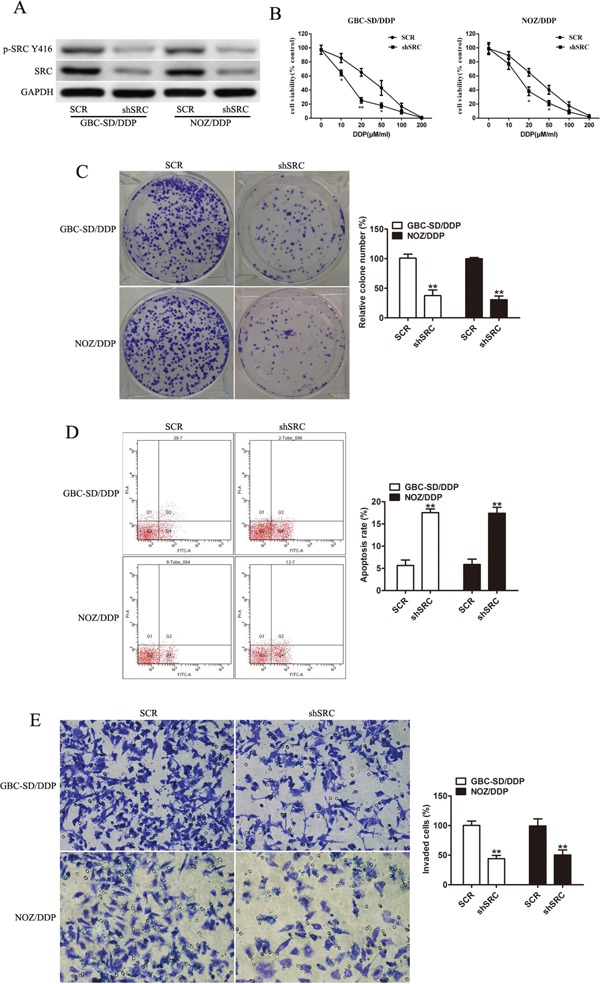
Loss of Src restores sensitivity to DDP and reduces the invasion capacity of GBC-SD/DDP and NOZ/DDP cells **A.** The protein levels of Src and p-Src(Y416) in GBC-SD/DDP and NOZ/DDP cells with shSrc transfection by Western blotting. GAPDH was used as an internal control. **B.** The DDP-sensitivity assay of GBC-SD/DDP and NOZ/DDP cells in the Src gene silencing group and the control group. **C.** The numbers of colony formation in the GBC-SD/DDP and NOZ/DDP cells with Src silencing after exposure to DDP. **D.** DDP-induced apoptosis evaluated in the GBC-SD/DDP and NOZ/DDP cells with or without Src silencing by flow cytometry. **E.** Cell invasion after DDP treatment evaluated in the GBC-SD/DDP and NOZ/DDP cells with or without Src silencing by Transwell assays. Data were presented as mean ± SD. *P< 0.05; **P< 0.01.

Since Src could be the direct target of miR-31, we further explored its functional relevance in DDP susceptibility with shRNA-mediated knockdown of Src. Src silencing in the GBC-SD/DDP and NOZ/DDP cells remarkably increased DDP sensitivity (Figure 4B), retarded cell proliferation (Figure 4C), promoted cell apoptosis (Figure 4D) and significantly suppressed cell invasion (Figure 4E).

### Enforced Src abrogates the function of miR-31

**Figure 5:**
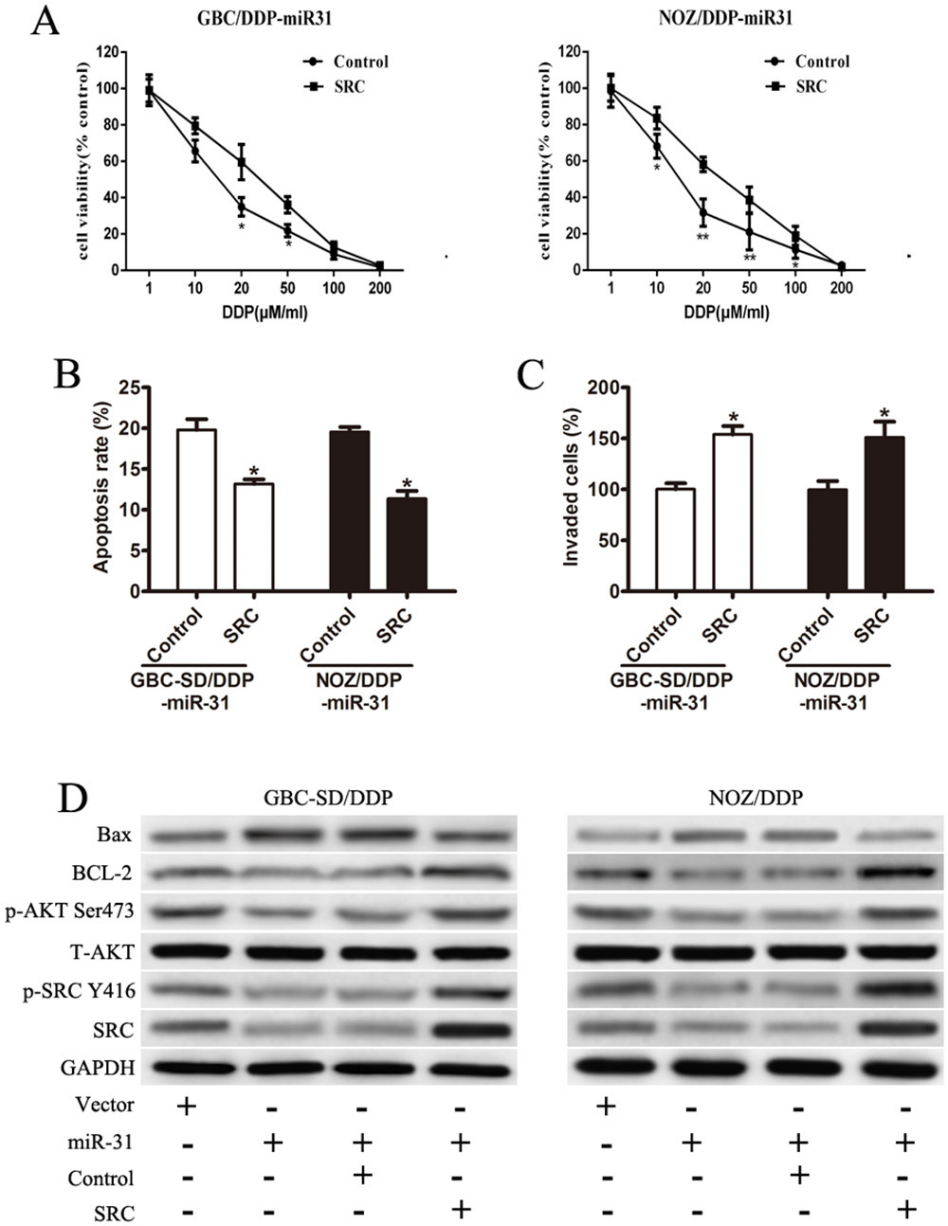
Enforced Src abrogates the function of miR-31 **A.** The restored drug-sensitivity in both GBC-SD/DDP-miR-31 and NOZ/DDP-miR-31 cells after Src reintroduction. **B.** The rate of DDP-induced apoptosis in both GBC-SD/DDP-miR-31 and NOZ/DDP-miR-31 cells after Src reintroduction detected by flow cytometry. **C.** Cell invasion assay after DDP treatment in both GBC-SD/DDP-miR-31 and NOZ/DDP-miR-31 cells after Src reintroduction detected by Transwell assays. **D.** The expression of Src, p-Src(Y416), T-Akt, p-Akt(Ser473), Bcl-2, and Bax in transfected cell lines by Western blot. Data were presented as mean ± SD. *P < 0.05; **P< 0.01.

The Src expression vector was introduced into miR-31 expressing GBC-SD/DDP and NOZ/DDP cells to test whether the effects of miR-31 in governing the responsiveness to DDP could be counteracted by restoration of Src expression. The drug sensitivity assay showed that Src reintroduction remarkably restored DDP-resistance (Figure 5A). A distinct decrease in the rate of apoptosis cells were also observed in flow cytometry (Figure 5B). The inhibitory function of miR-31 on cell invasion was also reversed in miR-31+/Src+ cells (Figure 5C).

The expression and activity of Akt, a major downstream kinase of Src, were examined. With no altered expression levels of total Akt, the activated p-Akt (Ser473) was clearly reduced after Src depletion in miR-31+/Src- cells, but restored in miR-31+/Src+ cells (Figure 5D). Furthermore, directly or indirectly phosphorylated by Akt, the balance of Bcl-2 family proteins were significantly disrupted, with decreased protein expression of Bcl-2 and increased expression of Bax in miR-31+/Src- cells (Figure 5D). These observations were reversed in miR-31+/Src+ cells (Figure 5D).

### MiR-31 increases chemosensitivity of NOZ/DDP cells *in vivo*

**Figure 6:**
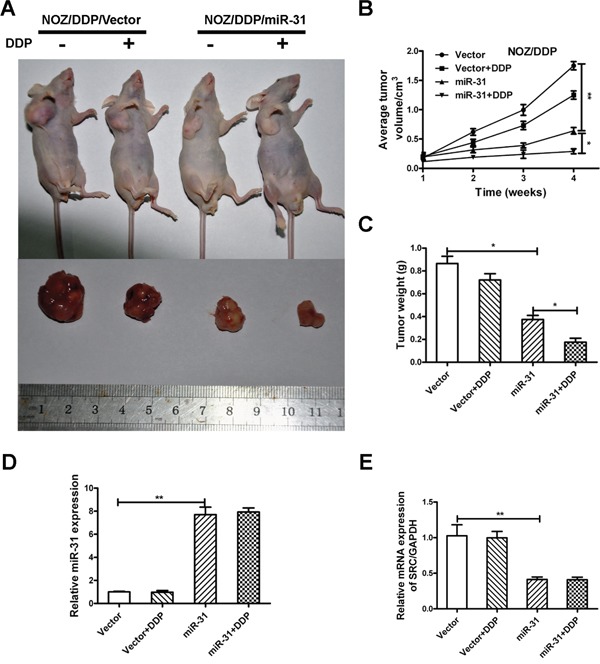
MiR-31 increases chemosensitivity of NOZ/DDP cells *in vivo* **A.** The xenograft tumors excised from the nude mice subcutaneously injected NOZ/DDP cells transfected with miR-31 or vector with or without treatment with DDP. **B.** The effect of miR-31 on the volume of xenograft tumors treated with DDP. **C.** The effect of miR-31 on the weight of xenograft tumors treated with DDP. **D, E.** The expression levels of miR-31and Src mRNA of xenograft tumors quantified by qRT-PCR. Data were presented as mean ± SD. *P < 0.05; **P < 0.01.

Given the crucial function of miR-31 *in vitro*, we investigated the link between upregulation of miR-31 and amelioration in the responsiveness to DDP in xenograft tumor models. The mice injected with NOZ/DDP-miR-31cells developed GBC tumors with smaller size (*P*<0.01) and lighter weight (*P*<0.05) than the control mice, and tumor volume and weight continued to decline when DDP was added (Figure 6A–6C). The qRT-PCR confirmed substantially elevated expression of miR-31 and reduced expression of Src in the tumors treated with miR-31expressing cells (Figure 6D, 6E).

## DISCUSSION

Individuals with GBC may have a poor prognosis due to GBC cells resistant to DDP-based chemotherapy regimens. Comprehensive researches have been made to explore the potential mechanisms of DDP-resistance [14]. A range of miRNAs have been found extensively involved in the carcinogenesis and chemoresistance [15]. Our study may be the first to demonstrate that miR-31 could decrease DDP-resistance by targeting Src in GBC.

Aberrant levels of miR-31 have been illustrated in various malignancies, such as high expression in colorectal cancer and pancreatic cancer [16, 17], low expression in breast cancer, ovarian cancer and prostate cancer [10, 11, 13]. MiR-31 is not only linked to tumor proliferation, invasion and metastasis, but also to the acquisition of onco-chemoresistance [10]. Wang et al showed that suppression of miR-31 increased sensitivity to 5-FU and inhibited cell migration and invasion in colon cancer [18]. Conversely, it was observed that miR-31 was downregulated in ovarian cancer cells that acquired paclitaxel (PTX) resistance, and re-introduction of miR-31 re-sensitized the cells to PTX [19]. In our study, miRNA microarray revealed that miR-31 was notably downregulated in DDP-resistant GBC cells. Ectopic over-expression of miR-31 promoted apoptosis and increased chemosensitivity to DDP both *in vitro* and *in vivo*. Upregulation of miR-31 was also found to sensitize advanced prostate cancer cells to apoptosis following exposure to DDP [20].

The ubiquitously expressed Src, a non-receptor tyrosine kinase, is the first discovered proto-oncogene function as a critical regulator in tumorigenesis and metastatic progression [21]. The overexpression and hyper-activation of Src have been found in a large variety of malignancies including GBC [22], and may confer resistance to chemotherapeutic agents such as 5-FU, doxorubicin and DDP [23–25]. We found that miR-31 may directly target the 3′-UTR of the Src mRNA based on a perfect match of binding sites detected by the Target Scan and miRBase databases. This was further verified by the findings that luciferase activity of the Src 3′UTR (wild-type) was significantly repressed after treatment with the miR-31 mimics. Moreover, an inverse correlation between miR-31 and Src mRNA expression was identified in both GBC cells and tissue samples. Additionally, Src ablation by RNA inference recapitulated the effect of miR-31overexpression, and exogenously overexpressed Src in miR-31 expressing cells restored the DDP-resistance. All these observations strongly indicated that miR-31 may directly target Src and downregulate the expression and function of Src.

The failure of chemotherapy-induced apoptosis may lead to chemoresistance [26]. Akt, as one of the downstream kinases of Src, has been implicated in anti-apoptotic effect by disrupting the balance of Bcl-2 family proteins [27, 28]. In our work, depletion of Src, accompanied by a decreased expression of activated p-Src (Y416), reduced the level of activated p-Akt (Ser473) without changes in total Akt expression level. The subsequently elevated expression of Bax and decreased expression of Bcl-2 strengthened DDP-induced apoptosis. In contrast, enforced Src attenuated the DDP-induced apoptosis in GBC with clear upregulation of Bcl-2 and downregulation of Bax. Collectively, the Src/Akt/Bax/Bcl-2 signaling cascade could be activated in the miR-31-downregulated DDP-resistant GBC cells, and downregulation of Src sensitized the miR-31 expressing GBC cells to DDP.

Our study is the first to reveal that expression of miR-31 is downregulated in DDP-resistant GBC, and miR-31may directly target and inhibit Src mRNA expression. MiR-31 may increase chemosensitivity of GBC cells to DDP both *in vitro* and *in vivo*, possibly through the Src/Akt/Bax/Bcl-2 signaling pathway, which may offer a therapeutic potential to overcome drug-resistance in GBC.

## MATERIALS AND METHODS

### Patient specimens and cell lines

The research was approved by Research and Ethics Committee at Xinhua Hospital, School of Medicine, Shanghai JiaoTong University, China. The written informed consent was obtained from all participants. The resected specimens were collected between January 2012 and January 2015 from 41 patients with GBC whose diagnosis was confirmed by two independent pathologists. The cohorts of cancer tissues and case-matched noncancerous tissues were established.

The human GBC cell lines (NOZ and GBC-SD) were obtained from the Cell Bank of Type Culture Collection of the Chinese Academy of Sciences (Shanghai, China). The NOZ and NOZ/DDP cells were grown in William's medium, while the GBC-SD and GBC-SD/DDP cells were cultured in Dulbecco's Modified Eagle's Medium (DMEM). Cisplatin -resistant GBC cells (NOZ/DDP and GBC-SD/DDP) were developed from NOZ and GBC-SD cell lines by treatment with gradually increasing concentrations of cisplatin in cell culture medium. Briefly, cells were seeded in six-well plates and reached about 80% confluency in fresh medium before treating with cisplatin. The dose of cisplatin range from 0.1 to 30 μmol/l and it was increased by a dose gradient that was 25–50% of the previous dose. The next dose was given until the cells were stable in proliferation without significant death.

### Transfection

The miR-31 expression vector, Src short hairpin RNA (shRNA), Src expression vector and their corresponding negative controls were designed by GenePharma (Shanghai, China). Inhibitor of miR-31 (anti-miR-31) were purchased from System Biosciences (SBI, USA). The GBC-SD/DDP and NOZ/DDP cells were seeded in six-well plates and transfected using Lipofectamine 2000(Invitrogen, USA) following the manufacturer's protocol.

### RNA isolation and qRT-PCR

Total RNA was extracted from tissues and cells using TRIzol Reagent (Invitrogen, CA, USA). To measure the level of miR-31, quantitative real-time PCR (qRT-PCR) was performed on an ABI 7900HT fast real-time PCR system (Applied Biosystems, FosterCity, CA, USA) according to TaqMan Small RNA Assays protocol. The endogenous U6 small nuclear RNA was used for normalization. To measure the level of Src mRNA, qRT-PCR was carried out using the SYBR-Green method (Takara). Glyceraldehyde 3-phosphate dehydrogenase (GAPDH) was used for normalization. Relative miR-31 and Src mRNA expression levels were calculated using the comparative threshold cycle (Ct) method. Designed by Sangon (Shanghai, China), the sequences of the primers used for amplification were as follows:

Src (forward): 5′-CAT CCA AGC CTC AGA CCC A-3′

Src (reverse): 5′-TGA CAC CAC GGC ATA CAG C-3′

GAPDH (forward): 5′-CAACAGCCTCAAGA TCATCAGC-3′;

GAPDH (reverse): 5′-TTCTAGACGGCAGGTCA GGTC-3′.

The results were normalized to endogenous GAPDH expression.

### Western blotting analysis

The treated GBC cell lines were harvested and lysed in RIPA buffer (Cell Signaling, Danvers, USA). The protein concentration determined by the bicinchoninic acid (BCA) protein reagent (Beyotime) Equal amounts of protein samples were run on the 10% SDS-PAGE and transferred onto polyvinylidene difluoride (PVDF) membranes. The membranes were probed with primary antibodies against human Src, p-Src(Y416), T-Akt, p-Akt(Ser473), Bcl-2, Bax, or GAPDH (Cell Signaling Technology, USA). After incubated with the secondary antibodies (HRP-conjugated goat anti-rabbit or goat anti-mouse IgG), the signal bands were visualized by enhanced chemiluminescence (ECL) western blotting detection reagent. GAPDH was served as the loading control.

### Colony forming assay

Upon transfection with miR-31mimics or shSrc, GBC-SD/DDP and NOZ/DDP, cells were trypsinized and plated into 6-well plates (700 cells/well) to form natural colonies, and treated with cisplatin (10 μM/ml) for 24 h before harvesting. After cultured for 10 days, colonies were washed twice with PBS, fixed with 4% methanol for 20 min, and stained with 1.0% crystal violet for 30 min. Then the colonies containing at least 50 cells were statistically analyzed and photographed. The experiment was repeated at least three times to ensure the data reproducibility.

### Cell viability assay

In the analysis of cell viability, the Cell Counting Kit-8 (CCK-8, Japan) assay was used. Briefly, the transfected and counted cells were seeded into 96-well culture plate (1000 cells/well) and treated with increasing concentrations of DDP ranging from 0 to 200μM/ml as indicated. After DDP-containing media was removed, the CCK-8 solutions were added and incubated for 3 hours. The optical density was measured at 450 nm with a microplate reader and illustrated as a percentage of the viability of control cells (% of control). All the experiments were performed in triplicate independently.

### Apoptosis assay and cell invasion assay

Stable miR-31, miR-31 plus shSRC or empty vector DDP-resistant cells (1 × 10^6^cells) were cultured in 60 mm dishes and treated with cisplatin (10 μM/ml) for 24 h before harvesting. Flow cytometry was performed using an Annexin V-FITC apoptosis detection kit (BD Biosciences, USA). 100 μL of 1× binding buffer containing 2.5μL of FITC-conjugated annexin-V and 1μL of PI (100μg/mL) was added to each tube and incubated at room temperature in the dark for 20 minutes, the apoptosis rate were determined using the flow cytometry (BD, San Diego, USA).

Matrigel-based Transwell chambers (BD Biosciences) were conducted to explore the capability of cell invasion. The equal numbers of transfected GBC cells were added into the top chambers. Following 48h incubation in serum-free medium, cells invaded through the Matrigel were fixed with methanol and stained with 0.1% crystal violet for 30 minutes, whereas cells remaining in the top chambers were removed using cotton swabs. Five microscopic fields were randomly chosen from each filter under the microscope, the average number of invaded cells were counted for quantification.

### Plasmid construction and lentivirus production

To construct the miR-31 expression vector, a DNA fragment containing miR-31 pre-miRNA was amplified from human genomic DNA and inserted into pCDH-CMV-MCS-EF1-copGFP lentiviral vector (SBI, Mountain View, CA, USA). LentiVirus packaging and infection were performed as previous described [14].

### Luciferase reporter assay

The 3′-UTR of Src was amplified by PCR and subcloned into the psi-CHECK2 luciferase reporter vector (Promega, Madison, WI). A psi-CHECK2 construct containing mutated bases on the predicted binding site of miR-31 was constructed using a QuikChange Site-Directed Mutagenesis Kit (Stratagene, Palo Alto, CA). All resulting constructs were verified by sequencing. In the luciferase reporter assay, the NOZ/DDP cells were co-transfected with miR-31 mimics and the resulting construct containing wild-type 3′UTR of Src (Src-WT) or Src 3′-UTR with mutation at the potential miR-31binding site (Src-MUT) using Lipofectamine2000 (Invitrogen, USA). According to the manufacturer's protocol, at 24 h post-incubation, the Renilla to firefly luciferase signal ratio was calculated for the quantification of relative luciferase activity with the Dual Luciferase Assay System (Promega, USA).

### miRNA microarray analysis

The total RNA from GBC-SD, NOZ and the DDP-resistant cells were harvested with Trizol reagent (Invitrogen, CA) and the RNA concentrations were tested by measuring the absorption value with a Nano Drop spectrophotometer. Having RNA samples labelled with the miRCURY^™^ Hy3^™^ /Hy5^™^ Array Power Labelling Kit and hybridized on the miRCURY^™^ LNA Array, the microarray assay was carried out. Subsequently, the Hybridization Station was utilized for hybridizing RNA samples with the spotted probes. The Axon GenePix 4000B Microarray Scanner was performed for signal scanning, and GenePix pro V6.0 was conducted for analyzing the collected hybridization images.

### Animal experiment

Male pathogen-free nude mice (6-week old, weighing 25–30 g, n=6) obtained from the Shanghai Laboratory Animal Center of the Chinese Academy of Sciences (Shanghai, China) were randomly divided into 4 groups. The animal experiments were approved by the Institutional Animal Care and Use Committee of Shanghai Jiao Tong University (Shanghai, China) and carried out in accordance with the experimental animal guidelines. Briefly, NOZ/DDP cells stably expressing miR-31 or vector were injected subcutaneously into the right axilla region of each mouse (5×10^6^ cells in 100 μl). Tumor sizes were measured using vernier caliper every two days when the tumors were apparently seen and tumor volume was calculated according to the formula: volume = 0.5 × Length × Width 2. Ten days after implantation, cisplatin (10 mg/Kg) was intraperitoneal injected in indicated mice. The mice were sacrificed at 4 weeks post-transplantation, and the xenograft tumors were removed, weighed and subjected to qRT-PCR analysis.

### Statistical analysis

SPSS software (version 19.0, USA) and GraphPad Prism software5.0 (GraphPad, USA) were used for all statistical analyses. In the quantitative measurements, the two-tailed Student's t-test was performed between the experimental groups and *p* <0.05was considered statistically significant. The measurement data are presented as the mean ± standard deviation (SD).

## SUPPLEMENTARY TABLE




