# Supplementary material for: MiR-31 regulates the cisplatin resistance by targeting Src in gallbladder cancer
Source: Oncotarget. 2016 Nov 4;7(50):83060–70. doi: 10.18632/oncotarget.13067 (PMC5347753; doi:10.18632/oncotarget.13067)
Supplement: Supplementary file 1 [file oncotarget-07-83060-s001.pdf]

MiR-31 regulates the cisplatin resistance by targeting Src in gallbladder cancer

SUPPLEMENTARY TABLE

Supplementary Table S1: The potential target genes of miR-31

See Supplementary File 1
